# Review of "Introduction to Biomedical Engineering, Second Edition", Edited by John Enderle, Susan Blanchard, and Joseph Bronzino

**DOI:** 10.1186/1475-925X-4-44

**Published:** 2005-07-19

**Authors:** Amit Gefen

**Affiliations:** 1 Department of Biomedical Engineering, Faculty of Engineering, Tel Aviv University, Tel Aviv 69978, Israel

The popular book by Drs. Enderle, Blanchard and Bronzino was now published in a second edition. The first edition, published on 1999, became the major textbook in many introductory courses given in the first or second year of Biomedical Engineering (BME) undergraduate programs. The second edition is aimed at serving the same purpose as the first one, i.e. to provide in depth and in breadth overview of the continuously growing field of BME. The dynamics of the field since the first edition was released is reflected in major changes in the second edition.

Specifically, the authors kept the division into two gross parts: (i) core biomedical engineering areas (chapters 4–10: biomechanics, rehabilitation, biomaterials, tissue engineering, bioinstrumentation, sensors and biosignal processing), and (ii) biomedical technology (chapters 12–17: modeling, genomics, computational biology, imaging, lasers and optics). The additions to part (ii), biomedical technology, are a chapter on genomics and bioinformatics (chapter 13, which replaces "biotechnology" in the first edition), and a chapter on computational biology and complexity (chapter 14). The new chapter on genomics (chapter 13) is motivated by the recent sequencing of the human genome (as well as numerous viruses, microbes, eukaryotes, yeast and rice). The second new chapter on computational biology and complexity (chapter 14) includes examples of cellular process models in individual cells, as well as in cell populations and systems. On the other hand, the texts on imaging were reduced and condensed, so that the technologies of ultrasound and MRI, each occupying a separate chapter in the first edition, are now surveyed under one chapter of "medical imaging".

The major parts (i, ii) follow background of basic anatomy, physiology and cell theory (chapter 1, which, as in the previous edition, serves a limited purpose of providing the terminology used in later chapters), and of moral and ethical issues (chapter 2). The chapter on moral and ethical issues had been extended and now includes practical sections on marketing medical devices in the US, and on the role of biomedical engineers in the process for FDA approval. Real and hypothetical case studies were also added here, to illustrate ethical issues, patient privacy concerns, and medical liability questions.

Overall, this remains an excellent textbook for BME students, and the progress in the field over the last 6 years is well reflected. Each chapter includes example problems with solutions and some 10–30 exercises. The list of suggested additional reading material, which concludes each chapter, was updated to cover literature published since the first edition was released. Figures are of good quality and are informative. Particularly useful is the new appendix on Matlab and Simulink software tools, which are required for solving some of the problems and exercises in this book. This not only contributes to the completeness, but also focuses the students on the computational abilities of these powerful software tools which are commonly used in BME work (e.g. solving polynomial and differential equations, plotting data, and simulating dynamic systems).

My only reservation relates to the level of mathematics and basic engineering sciences (e.g. solid and fluid mechanics, electrical circuit analysis etc.) which is expected from students in the tasks provided. Given that an introduction to BME course is offered in many undergraduate programs during the first year of studies, students may be frustrated by not having the necessary background. In my introduction to BME course at Tel Aviv University, Israel, which was based on the first edition, I had the impression that students do not take full advantage of what this book has to offer, simply because they did not yet study differential equations, numerical methods, statistics, solid and fluid mechanics, and electrical circuits. In BME programs where an introduction to BME course is offered in the second year of studies, this issue may be resolved, but often the motivation in teaching an introduction to BME course during the first year is to provide students with the "taste and flavor" of BME while they are dedicating most of their time to mathematics, physics, biology, and basic engineering science courses. The second edition does not solve this conflict.

In closure, despite the above reservation, this is certainly the most comprehensive textbook of its kind, and is recommended not only for undergraduate BME students but also for BME engineers in the industry or at the graduate level in the academia, as a reference book for a quick dive into new topics, or for an up-to-date survey of recent developments in this field.

